# Detached Placenta

**Published:** 1871-02

**Authors:** Geo. Abbott

**Affiliations:** White’s Corners, N. Y.


ART. II.—*Detached Placenta.* By GEO. ABBOTT, M. D., White's Corners, N. Y.

In the December number of the *New York Medical Record* are given two fatal cases of detached placenta, as reported to the New York Pathological Society, Oct. 26th and Nov. 9th, 1870, which forcibly reminded me of one, of which I took notes and made reflections, some years since, which I send you. If you deem it of sufficient interest and value, you are at liberty to publish it in your valuable *Journal.*

Friday, Sept. 5th, 1861. This evening, by invitation, I witnessed a Post Mortem, and received the following history from the attending physician:

Mrs. W——, aged 37, naturally robust, and general health considered good, being near full time in her fifth pregnancy, was taken at twelve last midnight with pains indicating the approach of labor, soon followed with flowing, sinking and coldness of the extremities.

An examination revealed the os uteri dilated only sufficient to admit the end of the index finger; he, therefore, at once applied the tampon, and commenced the administration of opium, cadmium and stimulants. The *pains were stopped,* the flooding partially stayed, and she rallied somewhat, only to sink again about eight o'clock, and die, undelivered, at eleven a. m.

The peritoneal cavity contained considerable blood-stained serum. The uterus was flaccid, though whole and distended by an apparently full termed fœtus; considerable congestion existed in the region of the ovaries and broad ligament; but as no rupture could be found, it was concluded that the bloody stain resulted from exosmosis. The fœtus occupied the right occipito iliac presentation, and, still in the membranes, it, with the placenta, was rolled out of the uterus, when the Doctor removed from the walls, a little below the fundus, what he at first took to be a ruptured placenta, but soon determined was a clot of blood, the full size of an afterbirth. The placenta was found in connection with the child and membranes; and, though closely examined, no abnormal condition of either it or the substance of the womb could be discovered.

Recapitulation.—Pains, quickly followed by flooding; os uteri dilated sufficient to admit the index finger; inserted tampon, ad ministered *opiates,* astringents and stimulants; *pains ceased,* flooding continued, patient sank and died. Cause of death: unstopped, accidental or concealed hemorrhage.

To improve by this experience we ask, what treatment is most judicious under similar circumstances? What are the indications?

Physiology answers, Close the open patulous mouths of the ruptured blood vessels; and prescribe astringents.

Mechanical philosophy reasons: Inasmuch as the uterus is a hollow, contractile viscus, only moderately distended with its fœtal contents, whose flaccidity admits of flowing from the open blood vessels and capillaries of its inner surface, and thence escape through the os externum, could its spongy, flaccid walls be strained tense and tight upon its contents, most of the blood vessels would be so closed by the pressure that the flooding must be very materially, if not wholly checked, and therefore advises the exhibition of active parturients, adding, lest dribbling from the capillaries might still jeopardize life, tampon the os; but demurs to the use of the vaginal tampon as inefficient, and likely, at the best, to leave much space above it for the accumulation of blood; therefore, calculated more to hide than check the flow; and suggests, as far more effective, the plugging of the os from the inside, which may be done by drawing off the amniotic fluid in which the fœtus floats, by which it, the fœtus, will be forced down and into the os, closing it effectively, somewhat like the valve of a pump, so long as uterine contraction is efficiently maintained.

Materia Medica admonishes that astringents are medicated or unmedicated, and applied internally or externally. That the medicated are taken internally, or applied externally to the seat of difficulty, which, in this instance, cannot be reached, and, therefore, must act by absorption, a process of slow operation; and that the unmedicated consist principally of cold, in the shape of evaporating lotions and freezing applications; that their action is rapid and powerfully revulsive, as well as somewhat parturient; and calls attention to ergot, as a most active and reliable parturient, capable of producing constant, powerful and unremitting contraction of the womb. That assafœtida, bromide of potassium, chloral, chloroform, etc., possess excellent anti-nervous properties. That stimulants, both permanent and diffusible, have a direct, exciting, and supportative action to the brain and heart. While opiates not only destroy sensibility, but are active and reliable *anti-parturients.*

Therapeutics takes the field, balances all in a twinkling, and commands: rupture the membranes, give heroic doses of ergot, firmly compress the bowels, and bathe them well with cold water; exhibit asafœtida, chloral, or a little chloroform, to allay the nervousness; and sustain the patient well with brandy and ammonia.

Let me continue. The first case to which I have alluded as being reported in the *Medical Record,* died undelivered, six hours from first pain, and within fifteen minutes after the first well defined symptoms of detached placenta was observed by her physician. The second was early diagnosed, and "the membranes ruptured as "the first step in the treatment, and administered ergot and *tea-* "*spoonful doses of laudanum* freely and frequently; and, though "the head descended, and seemed to fit against the *rigid fibers* of "the os so accurately that leakage would appear to be impossible,

"yet the bleeding continued—slight between the feeble pains, but "with very abundant gushes at each expulsive effort.

"Both physicians were reasonably certain of the diagnosis, fully "appreciated the appending danger, and were yet powerless on ac-"count of the non-dilation of the os, unable to introduce the for-"ceps without danger of rending the uterus. They still continued "to give ergot and *laudanum* in *heroic and frequent doses*." (The italics are mine.)

Thus the case was watched and cared for from 2 to 6.20 a. m., when further "counsel was called. At 7.30 her pulse at the wrists "were feeble, eyes turned back, face blanched, and in a word she "seemed entirely ensanguinated. The os was now sufficiently dilat-"ed to admit the cautious introduction of instruments. The forceps "were applied, and in less than fifteen minutes the woman was de-"livered. The placenta was found free, and its removal was fol-"lowed by a chamberful of dark grumous blood, evidently some-"time effused, followed by death some twenty-five minutes after "delivery." He says, "With the indications gathered from this "painful experience, I should not, in the future, place much de-"pendence upon the use of ergot in such cases, but at the *earliest* "*possible moment*, would dilate, and then turn or deliver with the "forceps," adding, finally, "*first* and *foremost* in the treatment of "this rapidly fatal complication, I should most certainly place "*manual interference*." (Italics are the reporters.)

Perhaps the objector will say, Well, there is your own case—membranes ruptured early—ergot in heroic doses—head fitted so accurately against the rigid fibers of the os, that leakage seemed impossible, yet did blood flow, and that too with abundant gushes, at each expulsive effort; and finally, patient died.

All this was to have been predicted, for the parturient effect of the ergot was completely neutralized by the more active ante-parturient power of the *frequent repeated teaspoonful doses of laudanum.*

By these doses, nature's efforts in her own behalf were materially crippled; and the excellent work so well begun by rupturing the membranes, became worse than nugatory—for the uterus, having thus been rendered feeble, flaccid and impotent for the exertion of any compression on the blood vessels; and the well fitting head allowing but little blood to escape externally, it must quietly yield to the accumulating blood, which soon fully occupies the place of the withdrawn waters, only to gush forth on the first effort at contraction; because the fœtus, being now circumstanced measurably as when in the amniotic fluid, the contractile or compressing force is first experienced by the fluid blood; and it rushes past the head and out before any considerable compressible effect is made upon the foetus to close it upon the os.

This communication is drawn out more extensively than was at first contemplated; for, on reflection, I felt that if error in theory and practice was being held up and propagated, to the endangering of human life, it was a duty we owe to mankind to investigate and expose, and, if possible, to correct the same. Therefore, though in a manner, I agree with the reporting physician, to wit, dilate and deliver at the earliest possible moment; I think he does not give eufficient prominence and appreciation to the iminent danger of death, before the hour of dilatation and delivery becomes possible. If life is to be spared at all, in this "rapidly fatal complication," it can hardly be by any other than the exercise of the most intelligent and judicious treatment; and I believe the time, and also the importance of the administration of ergot, and the *withholding of opiates*, is at an hour when "manual interference," further than the rupturing of the membranes, is impossible.

